# Supplementary figures and images for: IFNγ suppresses the expression of GFI1 and thereby inhibits Th2 cell proliferation
Source: PLoS One. 2021 Nov 22;16(11):e0260204. doi: 10.1371/journal.pone.0260204 (PMC8608330; doi:10.1371/journal.pone.0260204)

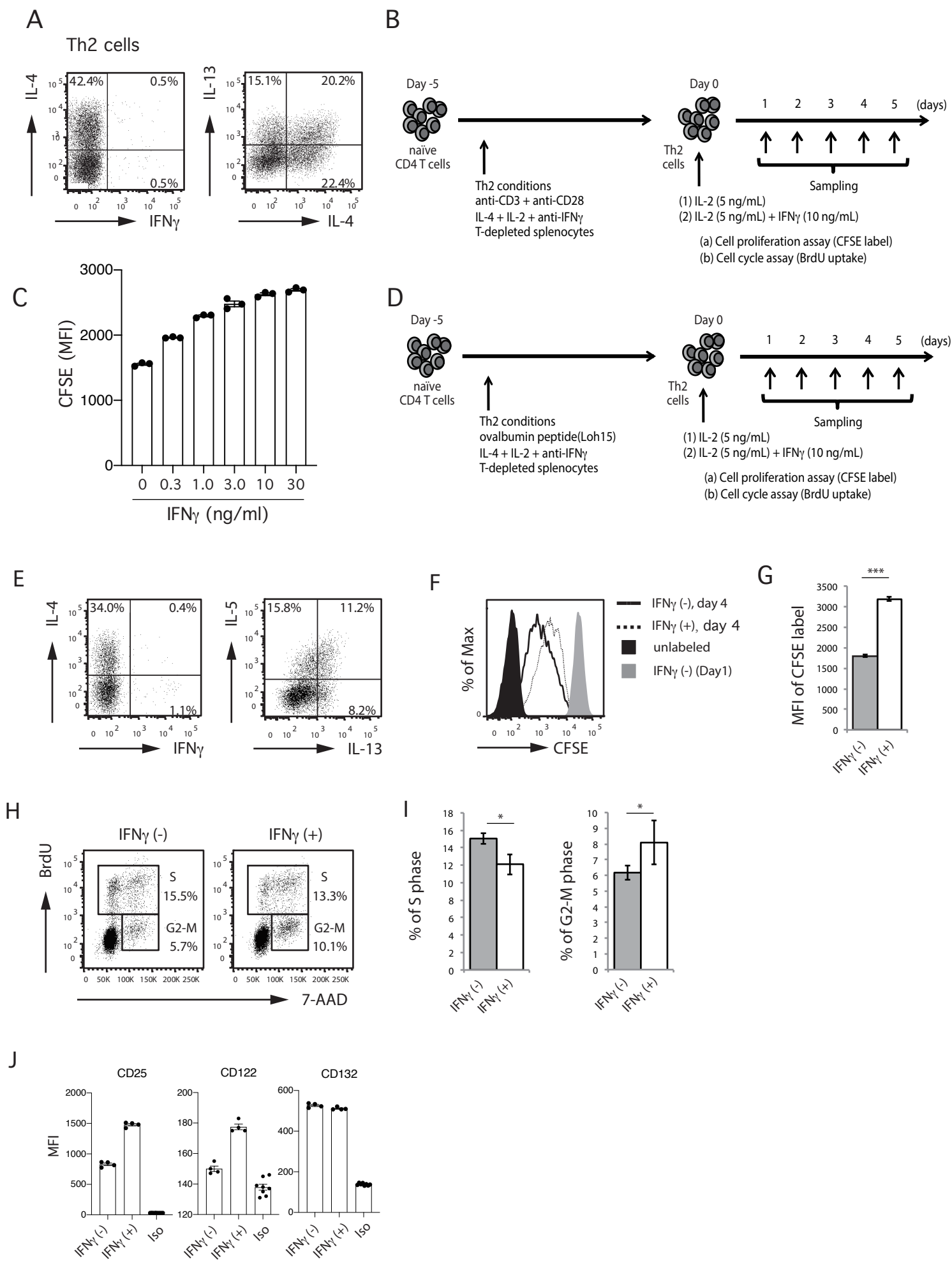

Supplementary Figure 1

Supplement: S1 Fig — (A) The cytokine production of Th2 cells that were in vitro-differentiated using polyclonal stimulations, as described in S1B Fig. (B) A schematic illustration of the experimental protocol using C57BL/6 mice. Naïve CD4 T cells were stimulated with soluble anti-CD3 and anti-CD28 Abs together with T-depleted splenocytes in the presence of IL-4, IL-2 and anti-IFNγ for 3 days, and further cultured with IL-2 for another 2 days to make fully differentiated Th2 cells. Cells were then labelled with CFSE, and further cultured with the indicated cytokines for the indicated number of days. (C) The MFI of CFSE dilution on cells cultured with the indicated concentration of IFNγ treatment for 4 days. (D) A schematic illustration of the experimental protocol using OTII TCR Tg mice. Naïve CD4 T cells were stimulated with OVA peptide (Loh15) together with T-depleted splenocytes in the presence of IL-4, IL-2 and anti-IFNγ for 3 days, and then further cultured with IL-2 for another 2 days to make fully differentiated Th2 cells. Cells were then labelled with CFSE, and further cultured with the indicated cytokines for the indicated number of days. (E) The cytokine production of Th2 cells that were in vitro-generated using OVA peptide stimulations as described in S1C Fig (F and G) A histogram (F) and the MFI (G) of CFSE on antigen-specific OTII TCR Tg Th2 cells cultured with IL-2 in the presence or absence of IFNγ for 4 days are shown. (H and I) A cell cycle analysis was performed using antigen-specific OTII TCR Tg Th2 cells after cultivation with IL-2 in the presence or absence of IFNγ for 4 days. (J) The MFI of CD25, CD122 and CD132 on Th2 cells cultured with or without IFNγ for 4 days is shown. Iso, isotype control. (PDF) [file pone.0260204.s001.pdf]

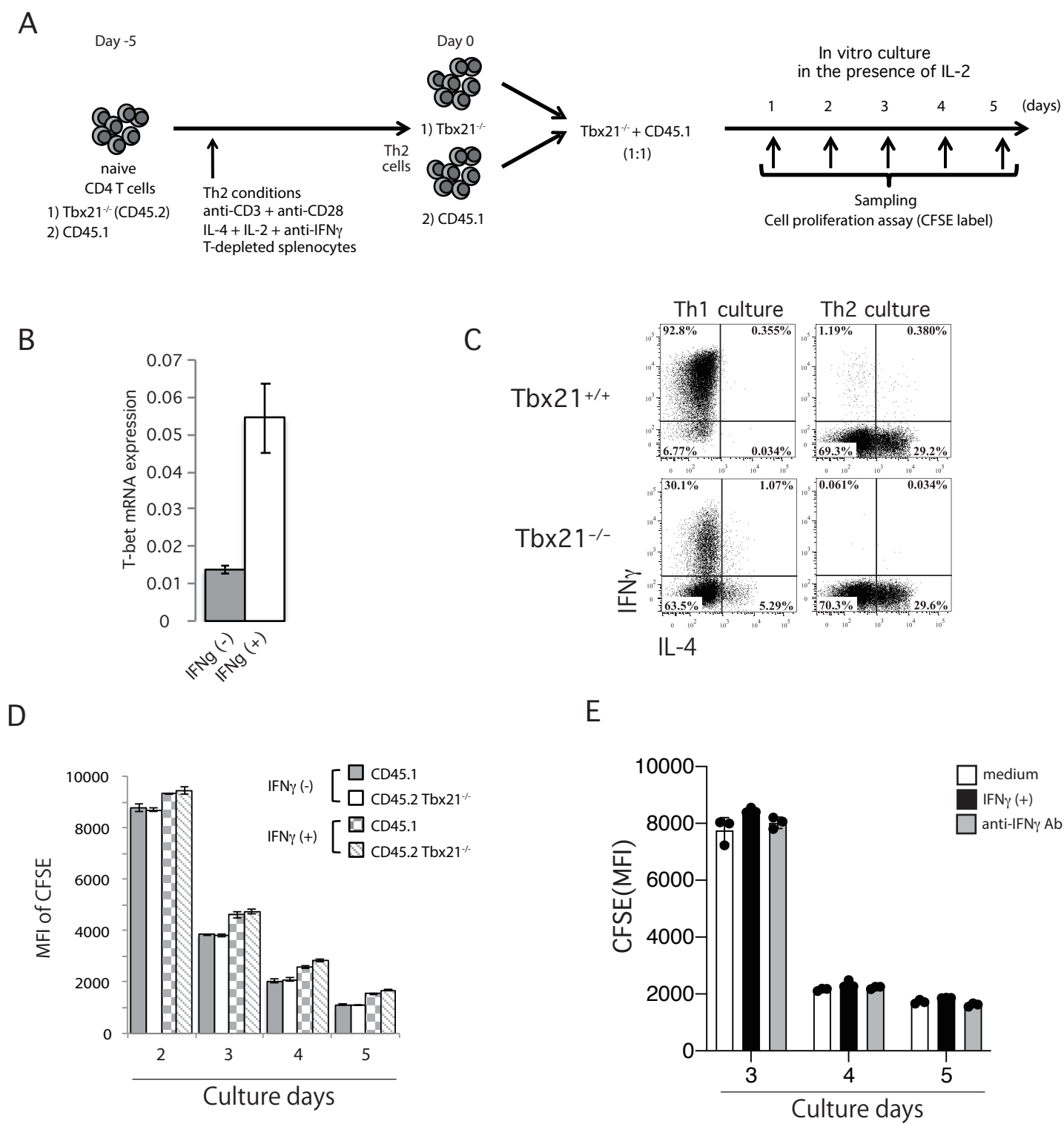

Supplementary Figure 2

Supplement: S2 Fig — (A) A schematic illustration of the experimental protocol using Tbx21-/- mice. Naïve CD4 T cells were isolated from Tbx21-/- (CD45.2), Tbx21+/+ (CD45.2) and congenic C57BL/6 (CD45.1) mice and cultured under Th2 conditions. Tbx21-/- Th2 cells were mixed together with CD45.1 Wt Th2 cells, labelled with CFSE and cultured with IL-2 in the presence of 4-OHT for the indicated number of days. (B) The Tbx21 mRNA expression relative to Hprt on Th2 cells after treatment with or without IFNγ. Error bars represent the mean ± SD. Data are representative of three independent experiments. (C) Intracellular staining of IL-4 and IFNγ production of Tbx21+/+ or Tbx21–/–CD4 T cells cultured for 5 days under Th1-skewing or Th2-skewing conditions is shown. (D) The MFI of CFSE on Th2 cells after cultivation for the indicated number of days. Data are representative of two independent experiments. (E) A flow cytometry analysis showing the MFI of CFSE dilution in Th1 cells after the cultivation with IFNγ or anti-IFNγ Ab for the indicated days. (PDF) [file pone.0260204.s002.pdf]

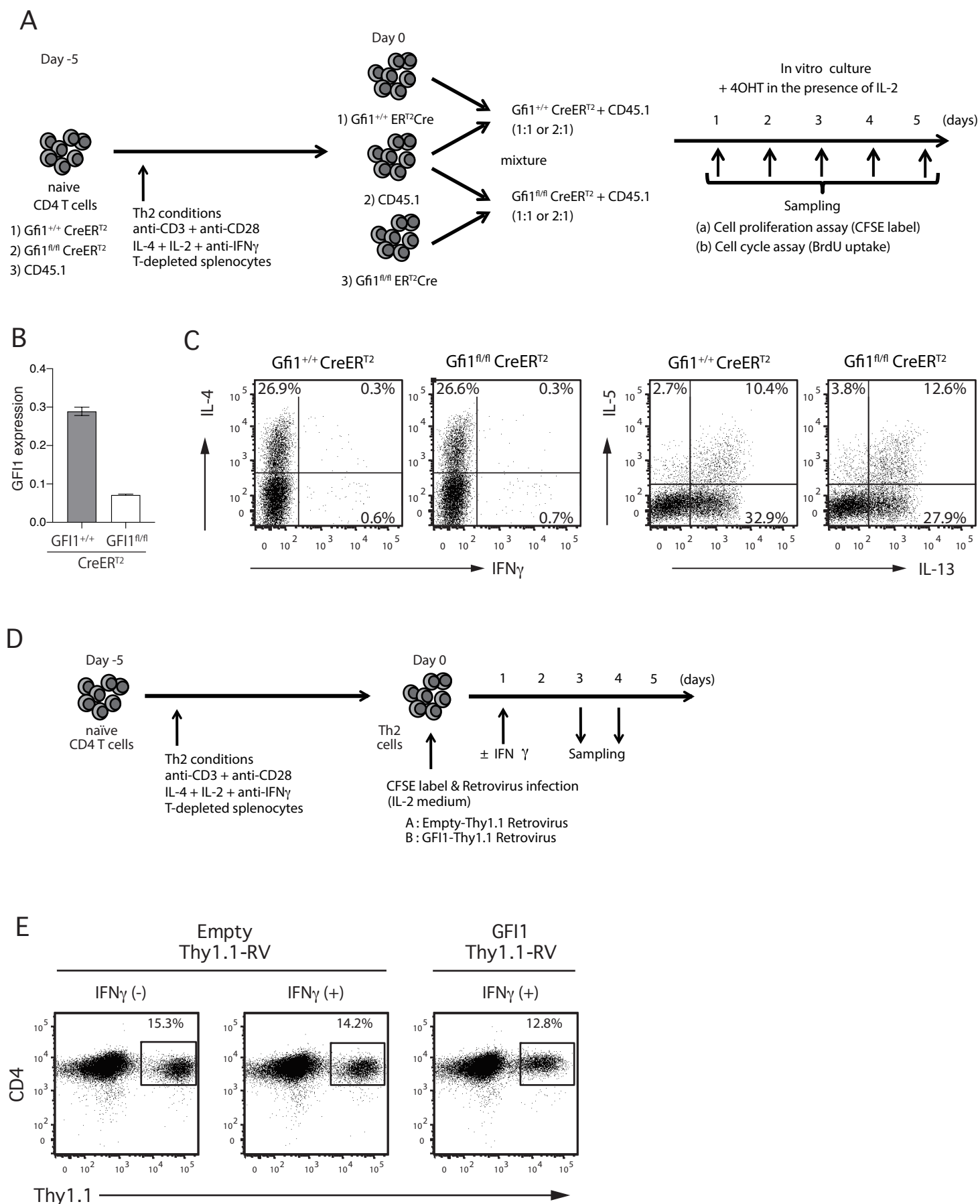

Supplementary Figure 4

Supplement: S4 Fig — (A) A schematic illustration of the experimental protocol. Naïve CD4 T cells were isolated from Gfi1fl/fl CreERT2 (CD45.2), Gfi1+/+ CreERT2 (CD45.2) and CD45.1 congenic mice and cultured under Th2 conditions. Gfi1fl/fl CreERT2 (KO) or Gfi1+/+ CreERT2 (WT) Th2 cells were mixed together with CD45.1 Wt Th2 cells, labelled with CFSE and further cultured with indicated cytokines (i.e., IL-2 and IFNγ) in the presence of 4-OHT for the indicated number of days. (B) GFI-1 mRNA expression relative to Hprt on cells after treatment of 4-OHT for 1 day. (C) The cytokine production of Th2 cells generated from the indicated mice as described in (A). Numbers indicate the percentage in each quadrant. Data are representative of three independent experiments. (D and E) A schematic illustration of the experimental protocol using retroviral infection. Naïve CD4 T cells were isolated from C57BL/6 mice and cultured under Th2 conditions for 5 days. In vitro-differentiated Th2 cells were then labelled with CFSE, infected with the GFI1-Thy1.1-retrovirus (RV) or empty-Thy1.1-RV and further cultured with IL-2 in the presence or absence of IFNγ. The MFI of CFSE was measured on day 3 or 4 by flow cytometry (D). The infection efficiency of Control-Thy1.1 RV and GFI1-Thy1.1 RV (E). (PDF) [file pone.0260204.s004.pdf]
