# Supplementary material for: IFNγ suppresses the expression of GFI1 and thereby inhibits Th2 cell proliferation
Source: PLoS One. 2021 Nov 22;16(11):e0260204. doi: 10.1371/journal.pone.0260204 (PMC8608330; doi:10.1371/journal.pone.0260204)
Supplement: S3 Fig — (A) In vitro-differentiated Th2 cells were further cultured in the presence or absence of IFNγ for 3 days. mRNA was prepared from these cells, and quantitative RT-PCR analysis of the indicated genes was performed. These gene expression changes are shown after being normalized with Hprt. The ratio of the gene expressions of IFNγ-treated cells relative to IFNγ-untreated cells) is shown. (B) A comparison of the relative expression (log2 values) of the selected genes between IFNγ-treated and untreated Th2 cells in (A). (PDF) [file pone.0260204.s003.pdf]

A

Gene symbol	-IFNγ	+IFNγ	ratio
Foxp3	0.402	0.131	0.33
GFI1	0.212	0.090	0.43
SOX4	0.091	0.039	0.43
GATA3	94.088	60.709	0.65
IRF4	0.270	0.189	0.70
Rorgt	0.194	0.142	0.73
Bcl6	0.171	0.152	0.89
IKAROS	2.407	2.176	0.90
Ink4a	0.316	0.294	0.93
IRF2	1.864	1.737	0.93
Tcf7	0.569	0.541	0.95
Egr3	0.070	0.068	0.97
Est1	19.306	19.567	1.01
IL-4ra	0.522	0.550	1.05
Prdm1	0.450	0.479	1.06
Egr1	0.092	0.101	1.11
Runx3	2.403	2.701	1.12
Batf	4.401	5.003	1.14
Hif1a	14.595	17.309	1.19
cMaf	9.245	11.000	1.19
Bcl11b	0.773	0.964	1.25
Est2	0.537	0.685	1.28
Id2	145.654	186.548	1.28
Rora	2.481	3.452	1.39
Runx1	0.427	0.602	1.41
cMyc	6.947	10.156	1.46
Id3	2.994	4.803	1.60
Nfil3	12.995	27.406	2.11
ROG	0.058	0.135	2.33
Rorc	0.038	0.097	2.53
IRF1	21.320	57.254	2.69
T-bet	0.302	0.922	3.05
Helios	0.037	0.128	3.48
Atf3	0.022	0.860	40.00
Eomes	ND	ND	
PLZF	ND	ND	
Spi-B	ND	ND	

qPCR normalization with Hprt

B

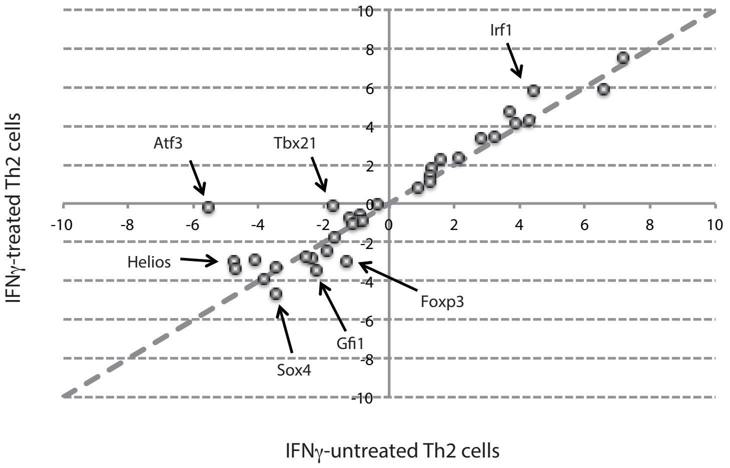

Supplementary Figure 3
